# Pericardial Effusion in Obstructive Sleep Apnea without Pulmonary Arterial Hypertension and Daily Hypoxemia - is it Unusual?

**DOI:** 10.4274/balkanmedj.2017.0580

**Published:** 2018-05-29

**Authors:** Emil Ivanov Manov, Ventsislava Pencheva Pencheva, Nikolay Margaritov Runev, Stefan Naydenov Naydenov, Daniela Stoychkova Petrova, Ognian Borisov Georgiev

**Affiliations:** 1 Department of Internal Diseases “Prof. St. Kirkovich”, Medical University of Sofia, Bulgaria

**Keywords:** Obesity, obstructive sleep apnea, pericardial effusion

## Abstract

**Background:**

Pericardial effusion in chronic hypoxemic lung diseases, such as Obstructive Sleep Apnea syndrome, usually occurs after the development of severe pulmonary arterial hypertension. However, data about the frequency of pericardial effusions in Obstructive Sleep Apnea syndrome without pulmonary arterial hypertension and/or daytime hypoxemia are still scarce, and their pathogenesis is unclear.

**Aims:**

To assess the prevalence of pericardial effusions and their volume and location in patients with obesity and Obstructive Sleep Apnea syndrome without pulmonary arterial hypertension and/or hypoxemia.

**Study Design:**

Cross-sectional study.

**Methods:**

We included 279 consecutive patients (162 males) with newly diagnosed Obstructive Sleep Apnea syndrome having a mean age of 42.8±12.4 years and a mean body mass index of 37.3±7.8 kg/m^2^. Obstructive Sleep Apnea syndrome was confirmed by polysomnography. Main exclusion criteria were concomitant inflammatory diseases, thyroid dysfunction, daytime hypoxemia, nephrotic syndrome, left ventricular systolic dysfunction and pulmonary arterial hypertension.

**Results:**

Pericardial effusion was found in 102 (36.56%) -all of them with moderate to severe obstructive Sleep Apnea syndrome. The mean effusion volume was mild to moderate (up to 250 mL). In 36 patients (35.3%) the pericardial effusion was diffuse, in 42 (41.2%), the pericardial effusion was located in front of the right atrium and the right ventricle, and in 24 (23.5%) the pericardial effusion was situated in front of the right cardiac cavities and the left atrium. We found a significant positive correlation between the presence of pericardial effusion and apnea-hypopnea index (r=0.374, p<0.001), body mass index (r=0.473, p<0.001), and desaturation time during sleep (r=0.289, p<0.001).

**Conclusion:**

Pericardial effusion in patients with obesity and moderate to severe Obstructive Sleep Apnea syndrome without daily hypoxemia and/or pulmonary hypertension is a relatively common finding. The occurrence of pericardial effusions is dependent mostly on the grade of Obstructive Sleep Apnea syndrome, degree of obesity, and duration of sleep desaturation.

Obstructive Sleep Apnea syndrome (OSAS) is a hypoxemic lung disease, characterized by a frequent cessation in breathing during sleep as a result of obstruction of the upper airways because of poor motor tone of the tongue and/or airway dilator muscles (1,2). The prevalence of OSAS is estimated to be 3%-7% in men and 2%-5% in women (2,3,4,5,6). Pericardial effusion (PE) in chronic hypoxemic lung diseases, including OSAS, usually occurs after the development of severe pulmonary arterial hypertension (PAH) (6,7,8,9,10,11). However, data about the presence of PE in OSAS without PAH and/or daytime hypoxemia are still scarce and unclear. This fact provided the rationale to assess the prevalence of PEs, and both their volume and location in patients with OSAS without PAH and/or daytime hypoxemia.

## MATERIALS AND METHODS

In our study, we included 279 consecutive patients (162 males, 58.1%) with newly diagnosed OSAS, having a mean age of 42.8±12.4 years and a mean body mass index (BMI) of 37.3±7.8 kg/m2. The study was performed over the period September 1, 2014-October 10, 2015. Informed consent was obtained from all participants before study inclusion. The study was approved by the local ethics committee, and all investigations were performed in accordance with the principles of the Declaration of Helsinki. One hundred and three (36.9%) patients with severe OSAS also had medical history of stage 1 arterial hypertension according to the European Society of Cardiology (ESC) criteria. Exclusion criteria were clinical, instrumental, and laboratory data for concomitant inflammatory diseases; thyroid dysfunction; presence of daytime hypoxemia and/or hypercapnia; Nephrotic syndrome and/or hypoproteinemia; left ventricular systolic dysfunction; and Doppler-sonographic data for PAH.

**Testing procedures:** In all patients, OSAS was confirmed by polysomnography (PSG) for at least 4 consecutive hours according to the accepted international recommendations. The diagnostic system included four electroencephalographic channels, right and left electrooculographic channels, one chin and four tibialis anterior electromyographic channels, finger pulse oximeter, strain gauges for thoraco-abdominal movements, one electrocardiographic lead, a nasal airflow (pressure cannula), a nasal thermistor, and a digital microphone for snoring detection. Polysomnographic recordings were scored in 30-second epochs for sleep, breathing, and oxygenation according to the standard criteria of the American Academy of Sleep Medicine (AASM). Obstructive sleep apnea was diagnosed when an apnea-hypopnea index (AHI) >5 per hour was registered. It was graded as mild (AHI 5-15), moderate (AHI 15-30), and severe (AHI >30) according to the criteria of the AASM (12). The time of desaturation was measured without the use of noninvasive mechanical ventilation.

**All patients underwent the following laboratory and instrumental investigations:** Complete blood count with differential, fibrinogen, erythrocyte sedimentation rate, C-reactive protein, free triiodothyronine and thyroxine, thyroid stimulating hormone, arterial blood gas analysis (BGA), polysomnographic examination, and echocardiography (echo) by two independent physicians according to the protocol of the ESC Working Group on Echocardiography. The systolic pulmonary arterial pressure was evaluated from the tricuspid systolic blood flow by continuous wave Doppler. The volume of PE was assessed quantitatively by M-mode measurement of the end-diastolic echo-free space between the two pericardial layers from the parasternal short-axis and long-axis views and the apical views as well. According to the presence or absence of PE, patients were divided into two groups: PE positive (PE/+/) and PE negative (PE/-/), respectively.

### Statistical analysis

Statistical analysis was performed using the SPSS statistical package, version 16.0 (SPSS Inc., Chicago, IL). Data were summarized by frequencies and percentages for categorical variables and by minimal, maximal, mean values, and standard deviation for continuous variables. For comparison of categorical variables, we used an independent χ2-test and a Fisher’s exact test. The normality of distribution of continuous data was assessed by a Shapiro-Wilk test. A t-test and an ANOVA were used for comparing parametric data whereas a Mann-Whitney U test and Kruskal-Wallis test were used for comparison of nonparametric data. Relationships between PE and AHI, BMI and duration of the desaturation time during PSG records were evaluated using the Pearson’s correlation coefficient. We used receiver operating characteristic analysis to define the sensitivity and specificity of the several parameters for the prediction of PE. All results were considered statistically significant for p values <0.05. The statistical power of our study was calculated post-hoc by free for public use software - Post-hoc Power Calculator (web address: http://clincalc.com/stats/power.aspx). Using this software, the statistical power of the study (i.e., the ability to detect a difference between two study groups) was calculated to be 99%-100%.

## RESULTS

PEs were found in 102 of 279 patients (36.6%) with obesity and OSAS, but without daytime hypoxemia and/or Doppler-sonographic data for PAH. Table 1 summarizes demographic and PSG data of PE/+/ and PE/-/ patients included in our study.

All hypertensive patients from the study population had adequate blood pressure (BP) control (mean systolic and diastolic BP was 136±3.8 and 84.0±5.2 mm Hg, respectively). Analyzed laboratory parameters of PE/+/ and PE/-/ patients did not differ statistically. We did not find significant differences in the evaluated structural and functional echo and Doppler parameters-there was not significant left ventricular hypertrophy (Table 2). All patients in both study groups had inspiratory collapse of the inferior vena cava >50%.

We also found no data for diastolic dysfunction in both groups–analyzed diastolic parameters were comparable for PE/+/ and PE/-/ patients (Table 3).

**Figure 1:**
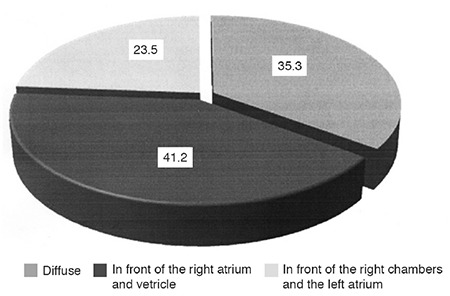
Location of pericardial effusions in analyzed patients with obstructive sleep apnea syndrome without pulmonary arterial hypertension and/or daytime hypoxemia.

The effusion volume measured in our patients was small with diastolic pericardial free-echo space 5-10 mm, corresponding to 150-250 mL of pericardial liquid. In 36 patients (35.3%) PE was diffuse, in 42 patients (41.2%); PE was located in front of the right atrium and the right ventricle, and in 24 patients (23.5%) PE was situated in front of the right cardiac cavities and the left atrium (Figure 1).

**Figure 2:**
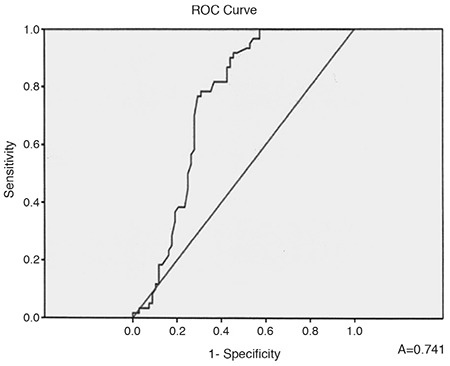
ROC-curve of apnea-hypopnea index for prediction of pericardial effusion.

The values of the mean AHI in the two groups showed a significant difference (Table 1), and a moderate positive correlation between the presence of PE and AHI was found as well (r=0.374, p<0.001). The calculated ROC-curve is shown in Figure 2. The area under the curve (AUC) was 0.741 [95% confidence interval (CI): 0.652-0.830], p<0.001.

**Figure 3:**
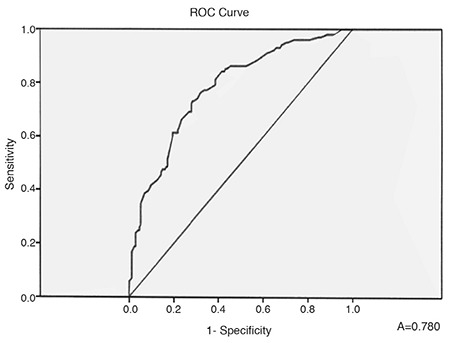
ROC-curve of body mass index for prediction of pericardial effusion.

We also found a statistically significant difference in BMI between the two groups (p=0.026, Table 1) and a statistically significant positive correlation between BMI and risk of development of PE (r=0.473, p<0.001). The ROC-curve of the BMI is presented in Figure 3. The AUC was 0.780 (95% CI: 0.723-0.836), p<0.001.

**Figure 4:**
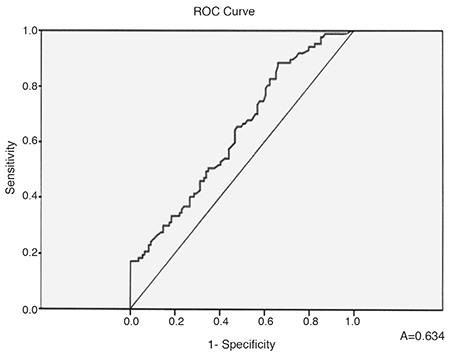
ROC-curve of desaturation time for prediction of pericardial effusion.

The desaturation time during the records was significantly prolonged (p=0.036), (Table 1) in PE/+/ patients with–a positive correlation between its value and the presence of PE (r=0.289, p<0.001). The calculated ROC-curve for the desaturation time is shown in Figure 4. The AUC was 0.634 (95% CI: 0.557-0.711), p<0.001.

## DISCUSSION

In our study, we found small to moderate PE in 36.6% of patients with newly diagnosed OSAS, but without daily hypoxemia from BGA and/or Doppler-sonographic data for PAH, i.e., the reported mechanisms for PE development in chronic hypoxemic lung diseases probably are not applicable to our patients. Peculiarly, we did not find a reliable explanation for the presence of PE in these cases in the published literature. It is important to note that even though PE was not detected in mild OSAS, it was identified in patients with moderate to severe OSAS without PAH and/or hypoxemia. Persistent PE is a common finding in patients with OSAS (9). Although such PE is usually not life-threatening (without cardiac pre-tamponade/tamponade), it could be very resistant to treatment (3,4,5,13). The exact mechanisms for the development of PE in many patients with OSAS are still uncertain. According to the published data, there is a correlation between the existing hypoxemia in chronic pulmonary diseases and the development of PE, which does not appear to be so dependent on the severity of the obstructive or restrictive ventilatory insufficiency, but on the systolic right ventricular pressure (3,4,5,7). PE in patients with chronic hypoxemic lung diseases usually represents the existence of PAH and advanced right heart failure, and is associated with a poor prognosis (5,6,7,11). Small or moderate PE has been found in up to 54% of these patients (13). Even for these cases, the physiology of the formation and removal of pericardial fluid is not completely understood. The fluid resorption is probably via an extensive venous circulation and a system of lymphatic plexuses, which are located in the subepicardial region of the heart and ultimately drain into the right atrium (5,6,7,8,9,10,11). A previous analysis by Raymond et al. (10) demonstrates that effusion size is correlated with right atrial (RA) pressure. Thus, it is likely that the PE observed in patients with PAH is due to both impaired venous and lymphatic drainage resulting from an elevated RA pressure (10). Interactions of the intrathoracic pressure changes (due to OSAS effects) and the RA pressure might be discussed, but we were not able to measure these possible variations in the RA pressure during apnea periods. Moreover, all our patients (with and without PE) had preserved inferior vena cava collapse (>50%) during inspiration. Using this practical approach for indirect evaluation of the mean RA pressure, we suggested the latter could not be steadily increased to explain the development of PE. Instead, our results demonstrated a positive correlation between AHI and PE. An apnea-hypopnea index of 31.90 had the highest predictive value for the existence of PE with sensitivity reaching 76.7% and specificity 70.6%. Our data also showed a positive correlation between the duration of sleep desaturation and the presence of PE, i.e., the longer the desaturation episodes, the higher the likelihood of PE. Desaturation time of 37.3 min. predicted concomitant PE with sensitivity 65.5% and specificity 52.3%. However, a statistically significant relation between PE and the lowest level of arterial O_2_ saturation during sleep in our patients was not detected. Published data on the occurrence of PE in obesity indicate the integral role of pulmonary embolism in the development of both PAH and right-sided heart failure (14,15,16). In our study we found a positive correlation between the presence of PE in patients without PAH and the severity of obesity with BMI of 37.1 kg/m^2^ having the highest sensitivity (73.3%) and specificity (71.0%). It is known that abdominal adipose tissue can increase levels of both systemic inflammation and oxidative stresses, which are critical factors linking obesity with its complications (15,17,18). However, we did not study these relations, i.e., a hypothesis could only be suggested about the possible association of PE in obese OSAS patients without PAH and/or hypoxemia and elevated systemic oxidative stress. The limitations of our study are as follows: (1) This study is not a longitudinal one and does not allow interpretation of prevalence and etiopathogenesis of PE among the entire population of OSAS patients; (2) Even though the estimated pulmonary artery pressure seemed normal, we did not do right heart catheterization for confirmation; (3) The exclusion of a concomitant inflammatory disease and Nephrotic syndrome was based on available prior medical documentation, lack of clinical symptoms, and pathological laboratory constellations for these conditions. However, no additional instrumental investigations (abdominal echo, computed tomography, magnetic resonance, etc.) were done. (4) Follow-up of echo findings after noninvasive mechanical ventilation of OSAS patients was not performed.

In conclusion, PEs in our patients with obesity and moderate to severe OSAS without daytime hypoxemia and/or Doppler data for PAH were relatively common. The most frequent location of PE was in front of the right cardiac chambers, followed by diffuse effusion in front of both atria and the right ventricle. The lack of echo data for pulmonary hypertension, as well as systolic and diastolic left ventricular dysfunction in our study suggested that the development of PE was less likely due to hemodynamic reasons. Instead, our data revealed that PEs were dependent mostly on the OSAS grade, degree of obesity, and duration of sleep desaturation. However, these findings and the presence of other factors and mechanisms for PE occurrence in such patients require both additional verification and validation by further research.

## Figures and Tables

**Table 1:**
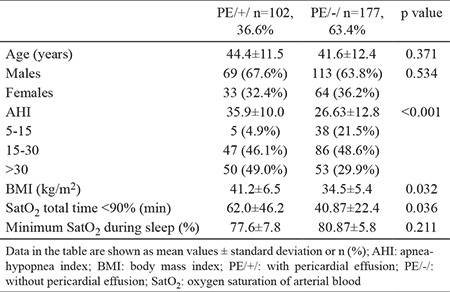
Demographic and polysomnographic data of the analyzed study population

**Table 2:**
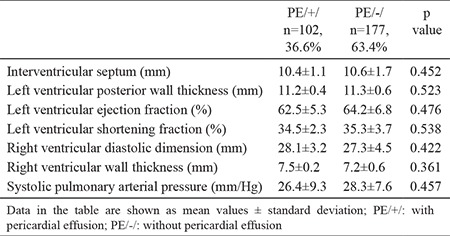
Echocardiographic and Doppler parameters of the analyzed study population

**Table 3:**
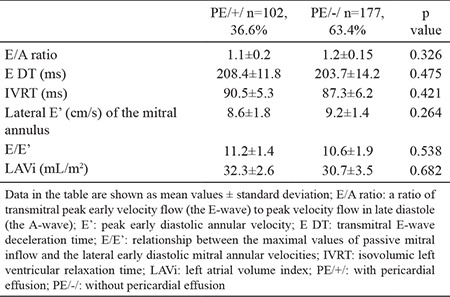
Doppler echocardiographic parameters of diastolic function in the analyzed study population
